# Mothers’ experiences of wellbeing and coping while living with rheumatoid arthritis: a qualitative study

**DOI:** 10.1186/s12905-022-01767-1

**Published:** 2022-05-19

**Authors:** Chloe Parton, Jane M. Ussher, Janette Perz

**Affiliations:** 1 School of Health, Te Herenga Waka - Victoria University of Wellington, Wellington, New Zealand; 2 Translational Health Research Institute, Western Sydney University, Locked Bag 1797, Penrith, NSW 2751 Australia

**Keywords:** Motherhood, Rheumatoid arthritis, Coping, Psychological distress, Subjectivity, Qualitative

## Abstract

**Background:**

Rheumatoid arthritis (RA) can result in difficulties for mothers when undertaking daily care activities and increased psychological distress. However, few studies have examined how women with RA subjectively experience coping and wellbeing as part of their motherhood.

**Methods:**

Twenty mothers with a diagnosis of RA and a dependent child (18 years or younger) who were living in Australia took part in a semi-structured interview between June and November 2017. Purposive sampling was undertaken to include participants across degree of current RA severity, number and age of children, and having received a diagnosis before or after a first child to take account of variability across these experiences. A qualitative thematic analysis was conducted on the interview transcripts.

**Results:**

The following themes were identified: ‘Burden and complexity in the mothering role’, ‘Losing control: Women’s experiences of distress’, and ‘Adjusting and letting go: Women’s experiences of wellbeing’. Experiences of distress, including feelings of failure, were associated with accounts of a loss of control over mothering practices among women, regardless of child age. In contrast, accounts of adjusting mothering practices and relinquishing control were associated with reports of enhanced wellbeing. In addition, some mothers reported greater ease due to increased independence of older children. The absence of social support exacerbated burden and distress in the women’s accounts, while the availability of support alleviated burden and was associated with reports of wellbeing.

**Conclusion:**

Health professionals and services can provide support to mothers with RA by addressing feelings of failure, acknowledging strategies of adjustment and letting go, and encouraging access to social support.

## Introduction

Rheumatoid arthritis (RA) affects 1.9 percent of Australians and approximately one percent of the population worldwide [1]. The condition has been found to have a significant impact on individuals’ lives and wellbeing through increased psychological distress, work place disability, lowered income and reduced ability to participate in social roles, including family relationships [2, 3]. Compared to men, women are diagnosed at a higher rate, are more likely to experience onset at a younger age and report more severe symptoms [4], including pain, joint stiffness and swelling, limited movement and fatigue [3]. Furthermore, symptoms and prognosis are unpredictable and people with RA report living with ongoing uncertainty around their daily functioning [5].

While the majority of people are diagnosed over the age of 50, many women with RA are of reproductive age [6]. Previous research has focused on pregnancy and reproductive outcomes [7], including the safety of pharmaceutical treatments during pregnancy and breastfeeding [8], family planning, pregnancy and postpartum experiences and information needs [9–11], as well as employment as a mother living with RA [12, 13]. Few studies have examined how women subjectively experience their motherhood while living with RA, including how they account for experiences of wellbeing and coping, across a range of child ages. This was the aim of the study.

No study has examined mothers’ subjective accounts of the coping strategies that they adopt as part of navigating experiences of distress and wellbeing with RA. There is limited evidence that RA can affect the performance of mothering roles due to pain, fatigue and impaired movement [13–15]. As part of coping with RA, women have reported altering daily care activities to accommodate physical limitations, including planning activities in advance to manage fatigue and having time out when in pain [16, 17]. Many of these alterations may be difficult to accomplish with younger children, due to their higher level of physical dependence on care [14]. Furthermore, the need to undertake these accommodations can have a detrimental influence on women’s subjectivities—their sense of self—as mothers [14, 17, 18], and can also result in distress [19]. In this vein, higher levels of physical parenting disability have been associated with greater levels of distress, particularly for women with younger children [19].

Mothering practices and subjectivities are influenced by a woman’s social and historical context, that informs experiences of motherhood and performance of a mothering role [20]. Accommodating actions of limiting contact or withdrawing from children because of RA are at odds with cultural expectations that women adopt more intensive styles of motherhood that are child-centred, emotionally absorbing, and time and labour intensive [21, 22]. According to these expectations, women are primarily and individually responsible for both their own and their child’s current and future wellbeing [23]. This can result in moral and psychological consequences for those who are not able to live up to idealised standards of the ‘good mother’ [20]. For example, there is limited research suggesting that women living with RA who report discrepancy between ‘ideal’ and ‘actual’ performance of their mothering role experience greater psychological distress [24]. This study addresses the need for further in-depth research to examine how women with RA negotiate cultural expectations of motherhood, including implications for women’s maternal subjectivities and wellbeing [25].

The aim of this study was to examine experiences of wellbeing and coping amongst mothers with RA across a range of child ages. We adopted a critical realist epistemology [26] and a material-discursive-intrapsychic theoretical framework [27]. This approach enabled us to recognise the materiality of the embodied impact of rheumatoid arthritis and women’s intrapsychic distress as being mediated by culture, language and power. Cultural constructions of gender and motherhood that privilege an idealised ‘good’ mother, and are used to make meaning of women and motherhood were particularly pertinent in this study [21, 22]. To address the research aim, the following research question was adopted: How do mothers with RA subjectively experience wellbeing and coping?

## Method

### Design

We adopted a qualitative study design. Twenty mothers with a diagnosis of RA and a dependent child (18 years or under) who were living in Australia took part in a semi-structured interview. The interview transcripts were analysed using thematic analysis to examine mothers’ subjective experiences of coping and wellbeing.

### Procedure

Recruitment was conducted through social media advertisements, including online support groups focused on topics of RA and motherhood. Advertisements asked for women with a diagnosis of RA and a dependent child (18 years or under) who were living in Australia to participate in a brief online survey on their experiences of being a mother while living with RA. At the end of the online survey, participants were invited to take part in a one-to-one interview to discuss their experiences in more depth. Interview participants were purposively sampled to across key factors to ensure that accounts of motherhood could be examined inclusive of variability across these experiences [28]. This included current severity of disease using self-report standardised measures of RA disease activity (Rheumatoid Arthritis Disease Activity Index, RADAI) [29] and impact (Rheumatoid Arthritis Impact of Disease, RAID) [30], diagnosis before or after having a child, and the number and age of children. Child age group was divided into younger (7 years and under) or older (8 to 18 years) to reflect the level of dependent care required by children in these groups. Rather than drawing on concepts of data or thematic saturation [31], our decisions around sample size were based on quality and richness of data generated through interviews, described as information power [32], as well as the number of interviews needed to account for variability in experience across demographic and health factors to generate novel insights into women’s experiences based on the research questions and epistemological position adopted [33].

All interviews were carried out by the first author (CP) who is trained in qualitative research methods and an experienced interviewer. Interviews were approximately an hour in length and conducted over the telephone for the convenience of women and due to geographic location. An interview guide was used that covered the following topic areas: how RA influenced the women’s experiences of motherhood and caring for children; self-care practices in which the women engaged; and experiences of formal and informal support, including prompts for support from partners, family, friends, and health professionals. As semi-structured interviews, a conversational approach was adopted with the wording and ordering of questions used flexibly to suit each participant interaction [34]. All interviews were digitally recorded and transcribed verbatim. All interview participants provided formal written or digitally recorded informed consent. The participants gave informed consent either via formal written or digitally recorded means depending on their preference. Ethics approval was provided through Western Sydney University Human Research Ethics Committee (H11500). All aspects of the research were conducted in accordance with the *National Statement on Ethical Conduct in Human Research 2007 (updated 2018)* [35].

### Analysis

Thematic analysis was used to analyse the interview transcripts [36, 37]. To conduct this analysis, transcripts were firstly read and re-read as part of establishing familiarity with the data. Initial coding was then undertaken by employing a ‘data-driven’, inductive approach to identify features of interest in the data that were relevant to the research questions. A coding framework was created by grouping initial codes around shared meanings to generate provisional themes. A line-by-line coding of the data was then conducted using NVivo to collate all instances of the provisional themes across the data set by the first author (CP), a process that was viewed as part of further familiarising all researchers with the data. ‘Higher order’ themes were then developed using word documents by authors CP and JU through a collaborative process of identifying patterns across the full coded data set, in relation to the research questions. Consistent with the epistemological position adopted [26], this involved a reflexive, reiterative process of organising data according to shared conceptual meanings that were informed by cultural constructions of gender and motherhood [21, 22], as well as the women’s accounts of lived experience of RA. The final analysis grouped coded data under the ‘higher order’ themes, ensuring internal similarity and distinction from other themes [38]. Differences that were noted in the mothers’ accounts according to child age group are described within the presented themes. Pseudonyms are used alongside the presentation of qualitative extracts in the results, in addition to participant age and child age group ("young" 7 years and under, or "older" 8 or more years) to provide context to the mothers’ accounts.

## Results

The 20 interview participants ranged in age from 23 to 54 years (average age of 36.6 years). Sixteen women were Anglo Australian (80%), three participants (15%) did not specify, and one identified as Latin American. The women had between 1 and 5 children (2 on average) across a range of dependent child ages. Fourteen of the women (70%) had a child 7 years or younger. Fourteen (70%) of the sample were in a relationship. Eighteen (90%) identified as heterosexual, with the remainder identifying as bisexual. The women reported mild to severe levels of current disease activity and impact against standardised measures. Scores ranged from 1.3 to 7.8 (M = 4.21, SD = 1.9) for disease activity (RADAI) and 0.0 to 9.88 (M = 5.14, SD = 2.11) for disease impact (RAID) from a maximum of 10 for both measures, with higher scores indicating greater disease activity and impact. Ten (50%) were engaged in full or part time employment, eight (40%) were not in paid employment, 2 (10%) women were currently studying.

The women’s accounts of subjective experience were complex, with participants often giving accounts of both wellbeing and distress while adopting a range of coping strategies. Overall, three main themes were identified in the analysis: ‘Burden and complexity in the mothering role’, including the subtheme, ‘Concerns about child wellbeing’; ‘Losing control: Women’s experiences of distress’, including the subtheme, ‘Taking back control over mothering at a cost to self’; and ‘Adjusting and letting go: Women’s experiences of wellbeing’.

### Burden and complexity in the mothering role

The women talked about their RA as a burden, which created additional complexity in their daily lives as mothers, due to the physical experiences of fatigue, pain, limited movement, and restrictions to the ability to lift or hold weight. For example, women reported challenges in navigating daily care activities including, holding and carrying young children, dressing children, changing nappies, bathing, transporting children, ensuring children’s physical safety, engaging in school events, supporting children’s interests and activities outside the home, and limitations to engagement in physical and outdoor activities. For example, Matilda (30 yrs, young child) said, “down to anything really like the clothes you dress her in because you don’t want anything too fiddly.” In addition, Denise (43 yrs, young children) described being unable to lift her baby to put her back to bed after night feeds. She said,

It was terrible. I mean she’s my baby, and I mean I felt terrible in the middle of the night not being able to put the poor little thing back to bed. And you know babies, they don’t really want anyone but their mum half the time.

Having older children was reported to be easier by some women as they were more “independent”, with Barbara (50 yrs, older children) saying, “I can do that little bit more now because they’re older”. However, other women commented that older children “want to do more things outside of the house” (Stephanie, 35 yrs, young and older children) and can come with their “own challenges … that mentally and emotionally can be quite draining” (Vanessa, 27 yrs, older child).

Rheumatoid arthritis was reported by many of the women to affect their “mood” and shape interactions with children. For example, women described having a “shorter fuse”, feeling “cranky”, or having “less patience” with their children when pain and fatigue were exacerbated. Ella (23 yrs, young children) described caring for her young children through a flare up, saying, “Just pure pain. Trying to do the things that I normally do just not to bore the kids. It just makes me crabbier and less tolerant.” Some women described withdrawing when pain and fatigue were exacerbated, in part to keep their responses towards children “under control” (Stephanie, 35 yrs, young and older children). For example, Holly (31 yrs, young and older children) said, “I’ll tell them to go away and I’ll explain to them why. I’d say, “Look I'm in a lot of pain. Just go away. I don’t wanna yell at you”. In addition, many of the women described avoiding touch to prevent exacerbating pain, which limited how the women were to hold, or engage in physical affection with their children. For example, Hilary (36 yrs, young and older children) described experiencing a flare up when her child was young, saying, “I think the physical closeness too wasn’t there because I couldn’t carry him. So, there were a lot of things that I just couldn’t do. So, I couldn’t lift my arm, I couldn’t walk around with him in a comforting way that I did with the first.”

Being a mother with RA was described as “very hard” by many participants, with symptoms shaping the “kind of mother” the women were. Many women reported that RA made them “a whole different parent” (Holly, 31 yrs, young and older children) who was less “hands-on” or “fun”. For example, women spoke about having limited opportunities to engage with their children in enjoyable physical activities, play, or to “take them places” due to their RA. Helen (36 yrs, young and older children) compared her current mothering experience to her life prior to RA, saying,

So, so different – it’s so different. When the other two were little, we used to go for walks and go to the park and we would just play, but now, especially being back at work, I find my pain levels are through the roof again and I’m just not fun mum.

Limitations to engagement in enjoyable and physical activities raised concerns for many of the women about how children might view them as mothers or reflect on their childhoods in the future, with Megan (38 yrs, young and older children) saying, “I do worry a little bit about how they are going to perceive me as a parent when they are older.”

#### Concerns about child wellbeing

Concerns were voiced by many of the women about how their RA might have a detrimental influence on their child’s wellbeing. This included concerns that children were “missing out” due to the women having less energy or not being able to engage with their children as they wished. For example, Adele (27 years, older child) said,

Sometimes I just – I end up falling asleep on the couch, and he’ll be watching a movie, or playing a game, or doing something. And I’ll fall asleep. And then – so, I feel like he’s kind of missing out on that interaction because I’m so run down.

Some women were concerned their children were experiencing distress, including “health anxiety”, due to witnessing their mother with RA, “sometimes you can’t help but cry out in pain when you’re trying to walk and things like that and it does scare him” (Thea, 36 yrs, young children). Many mothers reported feeling concerned their children might inherit RA. As Cathy (35 yrs, young children) said, “I have an underlying fear that they're gonna get it too”. Finally, some women also reported feeling concerned for older children who had taken on a caring role for their mother or siblings, due to fears this could limit their development. As Paula (42 yrs, older child) said,

It’s just frustrating when you can’t hold things and you can’t do things. She’s got to cut my food for me. So we’re planning on taking a trip to Bali, how am I going to cope getting on a plane, all these things. She’s got to get out and see places and do things.

In contrast, some women talked about their older children having additional “empathy” towards people with a disability, and “independence” due to the need to perform household tasks. Across these accounts, the women’s subjective experiences of motherhood were shaped by how they perceived their children to be affected by their RA.

### Losing control: Women’s experiences of psychological distress

Many women reported experiencing “frustration”, “sad[ness]”, “guilt”, “ang[er]”, and feeling “like a failure”, or “worthless” at being unable to do things for their families due to being a “sick” or “unwell” mother. For some women, this extended to reports of mental health difficulties, including “anxiety”, “depression”, or feeling “suicidal”. As Helen (36 years, young and older children) said, “I think I still expect me to be everything. So I just feel like because I can’t be, I just feel like I’m letting everybody down. I feel like I’m letting myself down.” Women talked about having less “confidence” and feeling like a “burden” on their families. For example, the following comments were made: “I just think I’m not as reliable a family member anymore” (Alison, 54 yrs, older child), and Thea (36 yrs, young children) said,

You know what, I hated myself. I hated my life. I felt very ungrateful because I had a beautiful son and a wonderful husband, and I wasn’t able to pull my weight. I still feel like a burden, to be honest, because essentially I am a burden on my family.

Many of the women reported not wanting to ask for help, to avoid feeling like a burden to their families and being a mother who could not cope independently. As Alex (33 yrs, young and older children) said, “I hate being reliant on anyone else”, and Stephanie (35 yrs, young and older children) said,

I struggle asking for help. In my head, I just feel that I should be able to do it all even though in reality, I know I can’t. It’s a constant battle mentally from reality to what you actually need.

Women also gave accounts of feeling “isolated”, with many reporting that they did not have people they could call on for support when needed. This was particularly evident in accounts from single mothers, women whose partners worked long hours or away from home, or women who lived away from wider family supports. For example, Holly (31 yrs, young and older children) spoke about her experience of being a single mother with RA, “I'm the only parent around to help. And I'm not even doing that very well half the time”. Similarly, Adele (27 yrs, older child) said, “sometimes, the help is just not available, so I don’t have time to be sick”. Some women also commented that because they “appear to be managing just fine” (Megan, 38 yrs, young and older child), they were less likely to receive support from others. In addition, due to fatigue a number of women spoke about not having the energy to maintain their own social relationships outside the home, meaning there were fewer people to call on when support was needed. As Thea (36 yrs, young children) said, “my life is very small isn’t it, just the cleaner, my husband, my son”. A lack of support to assist the women was talked about as adding to the burden of being a mother with RA.

#### Taking back control over mothering at a cost to self

Many women gave accounts of “pushing through” pain, discomfort and fatigue to meet their children’s needs without compromising expectations of themselves as mothers. For example, Vanessa (27 yrs, older child) spoke about engaging in physical activities with her child: “I do push myself and I do try. Sometimes I do suffer for the next few days because of that but I think it’s worth it”. Some women reported having higher expectations of themselves to make up for the impact of RA: “I probably put more expectations on myself. I think I actually probably try and make up for the fact that I’m not the parent that they always want me to be by doing more stuff” (Megan, 38 yrs, young and older children). However, many mothers also spoke about having no other option than to push themselves despite the physical effects of RA. For example, the following comments were made: “As a mum, you have to do it. You don’t have a choice.” (Stephanie, 35 yrs, young and older children) and “it’s a lot to look after yourself and someone else at the same time when you can’t physically stop vomiting or moving. You can’t—yeah. But—yeah, you don’t really have a choice” (Adele, 28 yrs older child). For mothers with RA, being unable to attend to self-needs could be detrimental to physical and emotional wellbeing, including longer term joint health. As Megan (38 yrs, young and older children) said, “it had quite dire consequences for me if I do it too much and too often I suppose”, and Holly (31 yrs, young and older children) said,

I just make it hurt and it’s gonna hurt because I need to get to where I'm going. I don’t like doing it. And my rheumatologist told me not to do it because that’s not good for your joints, but – yeah, nobody else is gonna drive me.

### Adjusting and letting go: Women’s experiences of wellbeing and coping

Many women also spoke about adjusting their mothering practices around their RA by altering the household environment, physical movements in daily tasks and activities, and arranging themselves alongside children when interacting and playing to avoid lifting or getting down on the floor. For example, women spoke about adjusting their environments so that “everything was at the right height for me” and limiting lifting young children. As Cathy (35 yrs, young children) said, “At home, I don’t do a lot of on the floor play. I would do—I put her up on the bed to play with her, saves me getting down on the ground—so, I guess it kind of—it impacts the way that I play with her.” Women also spoke about doing more “inside”, “quiet” and “sedentary” activities: “We have more activities at the couch (laugh) Like playing, like colouring, like reading, like playing with the iPad” (Amy, 37 yrs, young and older children). Other women spoke about being more organised and planning in advance to account for the unpredictability of RA, such as freezing meals in advance, “so that if it’s a bad day, I can heat them up” (Thea, 36 yrs, young children). For some women, adapting meant that they “end[ed] up operating in a completely different way”, which they did “without thinking” (Hilary, 36 yrs, young and older children). Furthermore, for some women, having “strategies in place” and adapting mothering practices meant that they were more “independent” and less reliant on support from others.

Children were reported to be positive motivators for some women to “keep going” and care for their own health and wellbeing. For example, Jodie (53 years, older child), said the following:

If I didn’t have a young child, I think I may have just curled up and died, because it was just too hard. It was too hard. Everything was too hard. Everything was painful. Everything was exhausting… she was every reason for me to just keep going. I had to get up.

In addition, Alison (54 years, older child) said, “I would’ve just stayed in bed. So, that’s really helped me actually because he was my reason to get up and get moving”. In contrast to accounts of taking control at a cost to self, women talked about being a mother as providing motivation to move and keep going, which had positive implications for self-care and joint health. For example, some women talked about prioritising their own self-care needs to preserve their health and wellbeing as part of being a mother. As Holly (31 yrs, young and older children) said, “since being sick and everything, I've realised it's okay to take care of yourself ‘cause if you take care of yourself, you can take care of the kids”.

Some women talked about lowering their expectations of themselves as mothers and acknowledging their limitations by doing less and prioritising other less physical aspects of their mothering role. For example, Vanessa (27 yrs, older child) spoke about doing less, saying, “I’ve decided that my house doesn’t need to be perfectly clear, the washing doesn’t always need to be done. So I’ve learned to really let that go and if I can’t do that stuff today, then I can’t do that stuff today, that’s just it.” Some women spoke about working in paid employment less, where financially possible, to “space it out”. For a couple of women, this meant prioritising material possessions less and focusing more on family relationships. As Amy said,

I noticed last week that I think I’ve been working actually more hours. So, I think I have to change job again. I have to be in some place where I can guarantee there can be more time with him, so rheumatoid has made me think about, okay how to present today or to think about – oh, I can do that tomorrow for him.

Across these accounts, women spoke about not being able to change their physical experience of RA, but adapting their “mindset”, lowering their self-expectations and prioritising time with children.

Finally, women gave accounts of seeking and accepting help from others that were associated with wellbeing. Having support was described as “the biggest thing” (Louise, 25 years, young children) that was critical for women’s coping (“I pretty much rely on grandparents and aunties and uncles to help me out”, Stephanie, 35 yrs, young and older children), and a way to create “balance” in their child’s life. For some women, having support from others was talked about as a normal part of motherhood, regardless of RA: “People say it takes a village to raise a kid and it definitely does” (Matilda, 30 yrs, young child). Accepting support from others was not without its challenges. For example, some mothers spoke about fathers engaging in play and physical activities with their children in lieu of their own participation, which some described as creating “pressure” for their partners. Additionally, Cathy (35 yrs, young children) spoke about some discomfort around her child bonding with another family member, saying, “[she] cried for her grandfather rather than me that time and that was a bit hard for me, like “no you’re supposed to want me.” In contrast to accounts of the absence of support, accounts of social support tended to function in general as a way to alleviate burden and create opportunities for greater balance for both mothers and their children.

## Discussion

This study provided insight into women’s subjective experience of coping as a mother while living with RA. Mothers gave reports of additional burden and complexity in their role due to RA regardless of child age. Accounts of the loss of control over mothering practices were associated with reports of distress, while reports of adapting or relinquishing control by lowering expectations were associated with accounts of wellbeing. Social support was described as alleviating burden, while lack of support was described as exacerbating the burden of mothering with RA across most of the women’s accounts.

Previous research found that mothers of young children report greater RA related parenting disability [19]. The women in this study reported experiencing burden and complexity in their role as a mother, and disruption of mothering expectations and practices, regardless of child age. Some women reported greater ease in their mothering role due to the increased independence of older children. However, many women with older children reported feeling concerned about their child’s adoption of a care role, which may have contributed to their feelings of failure and burden as a mother. These findings suggest that is important to consider women’s subjective experiences of mothering with a disability, including aspects of a mothering role that are pertinent to older children, such as emotional support and assisting access to activities outside the home. In general, the women’s qualitative accounts suggest that the emotional and relational aspects of mothering need to be acknowledged [25], in addition to physical difficulties.

Self-expectations as a mother are informed by cultural ideals that are unachievable for most women regardless of health status [39]. According to such cultural ideals, ‘good’ mothers are those who prioritise the needs of children over their own [40], utilising intensive styles of mothering in which women dedicate their time and emotional resources to the task of raising children [21, 22]. Furthermore, ‘good’ mothers cope independently, while perceived inability to cope is associated with a loss of control [41], and maternal subjectivities that are considered to be ‘lacking’ [25]. Congruent with a negative ‘lacking’ maternal subjectivity, the women in this study reported concerns that they were a ‘burden’ on their families, and that their children were ‘missing out’ due to the embodied impact of RA, with detrimental consequences for child wellbeing. Similar to previous studies with mothers in general [42, 43], accounts of discrepancy between self-expectations and actual experience were associated reports of distress by the mothers with RA, in response to a loss of control.

In contrast to mothers without RA, the women in this study positioned this discrepancy as being outside of their control due to their RA, while also reporting internalised feelings of failure, as has been reported by mothers with other forms of chronic illness, such as multiple sclerosis [44, 45]. The women’s accounts of taking back control over mothering at a cost to self are reminiscent of those suggesting that people with a disability engage in hidden physical and emotional labour to conceal their disability and protect others [46]. These findings highlight the cultural conditions that shape the complexity of mothering for mothers with RA, including women’s negotiation of the emotional and moral consequences of mothering practices when living with a chronic health condition [39, 47].

Similar to previous research on experiences of motherhood with RA, women reported adjusting mothering practices to accommodate their RA [16, 17], which was associated with accounts of wellbeing. In addition, the women reported lowering expectations by doing less while focussing on having time with children. Both practices can be interpreted as functioning to protect the women’s subjectivities as ‘good’ mothers [44]. By adjusting mothering practices, the women reported greater independence, enabling a sense of control. Letting go of expectations and prioritising time with children, can be interpreted as a way for the women to both resist idealised cultural expectations that were detrimental to their wellbeing [41], while also focussing on aspects of ‘good’ motherhood that they were able control, such as privileging time with children [21, 22].

Doing less is potentially risky for mothers with a health condition due to possibility of being labelled an ‘inadequate’ or ‘unfit’ mother [48]. It is important to note the women in this study associated doing less with enhanced wellbeing. Furthermore, while doing fewer physical activities, they also reported not compromising the relationship with their child. In contrast to accounts of a loss of control over mothering practices, RA was acknowledged and accommodated in these accounts, demonstrating the women’s agency in navigating the embodied impact of RA and cultural conditions of mothering, with implications for the women’s subjectivity as ‘good’ mothers.

Support served an important function across the women’s accounts of distress and wellbeing. Studies have found that social support is an important predictor of wellbeing for mothers in the general population [49, 50]. Individuals living with RA also report enhanced wellbeing to be associated with social support [51]. Comparably, the women in this study gave mostly positive accounts of support, as something that was ‘normal’ for good child raising or that created ‘balance’ for both themselves and their children. However, there were material and social conditions around availability and appropriateness of support in many of the women’s accounts. Firstly, a lack of available support was reported in qualitative accounts, particularly by women who were single, or whose partners had limited availability due to work commitments. In general, individuals with RA report greater social isolation [52], and lower household incomes due to decreased capacity for paid employment [3], which may contribute to increased work hours among partners, limiting women’s access to support.

The women’s accounts in this study also point to social support needs that are particular to the experience of mothering. Similar to research with mothers with other chronic health conditions [53], some women reported discomfort with support that was contrary to a ‘good’ mother role due to threats to primary attachment, or perceived as creating additional ‘burden’ or ‘pressure’ on family members. The tension between accessing appropriate support and the emphasis within culturally idealised motherhood on coping independently, has been described as creating a “tightrope” between independence and dependence for mothers who live with a disability [54]. Similarly, the findings in this study highlight the tensions negotiated by mothers living with RA when accessing appropriate support.

The strengths of this study include use of in-depth subjective accounts to examine how mothers living with RA negotiate their motherhood, coping and wellbeing across a range of child ages. As a limitation of the study, the sample was comprised of women who were predominantly heterosexual and Anglo Australian. Future studies could examine how women across sexual identities and diverse cultural groups negotiate coping and wellbeing while living with RA. For example, gendered expectations of parenthood shape the way parenting is negotiated within relationships [55], with same-sex couples reporting more equitable distribution of parenting tasks than heterosexual couples [56].

In future, studies could examine how heterosexual and same-sex couples negotiate parenting expectations and practices in the context of disability and illness. In addition, socioeconomic factors can shape how RA is experienced and managed. For example, reduced access to healthcare resulting in delayed diagnosis and access to medications [57]. Future studies could also address how socioeconomic conditions shape women’s mothering experiences, including the possibilities for women to adopt strategies and supports that enhance their coping and wellbeing as a mother. Finally, future research could examine how social support might influence experiences of distress, coping and wellbeing for mothers with RA. This includes barriers that women may experience to accessing support from their social relationships, as well as the negotiation of support within wider family relationships such as grandparent care [58].

The findings from this study have implications for the way that health professionals provide support for mothers living with RA. This includes acknowledging the importance of motherhood for many women with RA when addressing embodied impact of the condition for wellbeing. Furthermore, healthcare professionals could address mothers’ experiences of distress by challenging the idealised cultural ideal of ‘good’ mothering, and encouraging women to lower self-expectations where possible and privilege self-care, strategies demonstrated to be effective when working with vulnerable women [25, 59]. Finally, the material and cultural conditions which limit women’s access to social support need to be addressed, including normalising and encouraging women to access support where possible. The findings from this study suggest that mothers who live with rheumatoid arthritis experience additional burden and complexity in their role. Women can be seen to manage both risk to a ‘good’ maternal subjectivity, with their own RA management concerns, highlighting the complex negotiation of coping and wellbeing for women living with RA.

## Data Availability

Not applicable—data is presented in the article.

## Abbreviations

RA: Rheumatoid arthritis
AIHW: Australian Institute of Health and Welfare
NHMRC: National Health and Medical Research Council
ARC: Australian Research Council
